# Assessment of Fatty Acids Profile and Omega-3 Polyunsaturated Fatty Acid Production by the Oleaginous Marine Thraustochytrid *Aurantiochytrium* sp. T66 Cultivated on Volatile Fatty Acids

**DOI:** 10.3390/biom10050694

**Published:** 2020-04-29

**Authors:** Alok Patel, Ulrika Rova, Paul Christakopoulos, Leonidas Matsakas

**Affiliations:** Biochemical Process Engineering, Division of Chemical Engineering, Department of Civil, Environmental, and Natural Resources Engineering, Luleå University of Technology, SE-971 87 Luleå, Sweden; alok.kumar.patel@ltu.se (A.P.); ulrika.rova@ltu.se (U.R.); paul.christakopoulos@ltu.se (P.C.)

**Keywords:** oleaginous thraustochytrids, polyunsaturated fatty acids, volatile fatty acids

## Abstract

Thraustochytrids are considered natural producers of omega-3 fatty acids as they can synthesize up to 70% docosahexaenoic acids (DHA) of total lipids. However, commercial and sustainable production of microbial DHA is limited by elevated cost of carbon substrates for thraustochytrids cultivation. This problem can be addressed by utilizing low-cost renewable substrates. In the present study, growth, lipid accumulation and fatty acid profiles of the marine thraustochytrid *Aurantiochytrium* sp*. T66* (ATCC-PRA-276) cultivated on volatile fatty acids (C1, formic acid; C2, acetic acid; C3, propionic acid; C4, butyric acid; C5, valeric acid and C6, caproic acid) and glucose as control were evaluated for the first time. This strain showed an inability to utilize C3, C5 and C6 as a substrate when provided at >2 g/L, while efficiently utilizing C2 and C4 up to 40 g/L. The highest cell dry weight (12.35 g/L) and total lipid concentration (6.59 g/L) were attained when this strain was cultivated on 40 g/L of butyric acid, followed by cultivation on glucose (11.87 g/L and 5.34 g/L, respectively) and acetic acid (8.70 g/L and 3.43 g/L, respectively). With 40 g/L butyric acid, the maximum docosahexaenoic acid content was 2.81 g/L, corresponding to 42.63% w/w of total lipids and a yield of 0.23 g/g_cell dry weight (CDW)_. This marine oleaginous microorganism showed an elevated potential for polyunsaturated fatty acids production at higher acetic and butyric acid concentrations than previously reported. Moreover, fluorescence microscopy revealed that growth on butyric acid caused cell size to increase to 45 µm, one of the largest values reported for oleaginous microorganisms, as well as the presence of numerous tiny lipid droplets.

## 1. Introduction

Omega-3 and omega-6 fatty acids are considered essential fatty acids in the human diet because humans are unable to synthesize them in sufficient amounts [1]. Biosynthesis of eicosapentaenoic acid (C20:5n−3; EPA), docosapentaenoic acid (C22:5n−3, DPA) and docosahexaenoic acid (C22:6n−3, DHA) occurs from the parent omega-3 fatty acid α-linolenic acid (C18:3n−3), however, the conversion rates are too low to meet daily requirements [2,3]. Therefore, it is recommended to consume EPA and DHA through the diet. DHA plays an important role in the function and responsiveness of cell membranes, tissue metabolism and hormonal and other signaling pathways [4,5]. It also constitutes the major fatty acid in the brain and retina [6]. Significant quantities of DHA need to be supplemented by the diet during early brain development due to rapid neurogenesis [6,7]. DHA is the main fatty acid in the brain’s gray matter and its insufficiency has been linked to several major depressive and bipolar disorders, Alzheimer’s disease, schizophrenia and other types of dementia [8]. Notably, increasing omega-3 fatty acids (EPA and DHA) intake reduces also the risk of cardiovascular disease [9]. Randomized controlled trials have revealed that intake of fish and fish oil reduces the risk of fatal myocardial infarction (heart attack) and coronary heart disease mortality and may reduce the complications derived from elevated serum triacylglycerol in individuals with type-2 diabetes [10,11]. Marine fish belonging to the Salmonidae, Scombridae and Clupeidae families are an important source of omega-3 fatty acids, but, due to increasing demand for these lipids and a diminishing aquatic ecosystem, they are no longer a sustainable source of omega-3 polyunsaturated fatty acids (PUFAs) [12]. Plants can produce certain PUFAs, such as linolenic acid (18:2n−6), γ-linolenic acid (18:3n−6), α-linolenic acid (18:3n−3) and octadecatetraenoic acid (18:4n−3), [13,14] but they lack essential enzymes for the biosynthesis of EPA and DHA [15,16]. Finally, increasing concerns about the presence of contaminants in fish, which renders fish oil unsuitable for some applications such as infant formula, has led the quest for alternative sources of DHA [17].

Oleaginous thraustochytrid promise a potentially sustainable option for the production of microbial omega-3 fatty acids [18]. Thraustochytrids, a group of marine protists belonging to the Labyrinthula class of the Chromista kingdom, can grow heterotrophically and can synthesize high amounts of DHA. These properties make them excellent candidates for commercial DHA production, with the *Aurantiochytrium*/*Schizochytrium* genera being of particular interest [19]. In 2012, the global market for microbial DHA was estimated to be nearly $350 million and the value was revised up to nearly USD $4212 million in 2017, indicating a clear spike in demand for superior-quality microbial DHA [20,21,22]. Despite the advantages offered by heterotrophic production of DHA by oleaginous microorganisms, a major challenge is posed by the elevated cost of cultivation substrates, such as glucose, organic acids, yeast extract and corn steep liquor [22]. The carbon source (i.e., refined glucose) alone accounts for almost 80% of the total cost of cultivation media. This makes the overall production cost of microbial DHA highly dependent on the price of the carbon source and higher than fish oil [23]. Due to a continuous rise in the price of refined sugar over the past years, efforts have been made to explore alternative, less expensive carbon sources for DHA production [24,25]. Some studies have focused on the use of feedstocks from renewable and waste sources [26]. In this respect, biologic hydrogen production through dark fermentation under anaerobic conditions has attracted particular attention due to the simultaneous production of other valuable secondary metabolites know as volatile fatty acids (VFAs). VFAs contain six or fewer carbon chain fatty acids that can be easily distilled at atmospheric pressure. VFAs can be applied for the production of biofuels [27] and bioplastic [28]. In recent years, commercial-scale production of VFAs has favored the biologic route over chemical synthesis, as it allows the use of renewable substrates [29,30]. Nevertheless, because in the biologic route pure sources of sugar, such as glucose and sucrose, are utilized for the production of VFAs [31], this raises ethical issue about the utilization of food for chemicals. This concern can be eliminated by employing organic-rich waste materials, such as sludge derived from food waste, municipal solid waste and industrial water, for VFA production.

Several reports have been published to show the capability of heterotrophic freshwater microalgae to utilize VFAs as substrate, but they can assimilate only small quantity of VFAs (up to 2 g/L) as substrate [32]. Oleaginous yeast hardly utilizes more than 5 g/L of VFAs and showed an inhibitory effect on growth [33,34]. Only certain marine microalgae can utilize high concentration of VFAs [35]. In our previous reports, several species of thraustochytrids such as *Aurantiochytrium* sp. and *Schizochytrium limacinum* SR21 were cultivated for the production of DHA on either pure glucose or glucose derived from renewable substrates or waste stream [18,36].

However, marine thraustochytrid *Aurantiochytrium* sp. T66 (ATCC-PRA-276) was explored for the first time to cultivate on VFAs. The aim of the present work was to evaluate the ability of the marine thraustochytrid *Aurantiochytrium* sp. to utilize a range of different VFAs for growth and lipid production. Moreover, the effect of the different VFAs on fatty acid composition and particularly on DHA content was also studied. By understanding which specific VFAs promote high growth and DHA production, it will be possible to fine-tune the dark fermentation process and deliver a mixture of VFAs suitable for DHA production. Ultimately, the current work aims to give additional value to the traditional bio-hydrogen production process, by providing a nutraceutical compound (DHA) alongside bioenergy (hydrogen), while simultaneously treating organic waste streams.

## 2. Materials and Methods

### 2.1. Microorganism and Cultivation

The marine thraustochytrid *Aurantiochytrium* sp. T66 (ATCC-PRA-276) was procured from the ATCC. It was initially cultured on ATCC^®^ 790 By + medium containing yeast extract (1 g/L), peptone (1 g/L), glucose (5 g/L) and seawater (1000 mL) at 25 °C with 180 rpm for 48 h. After that, the thraustochytrid was cultivated at 25 °C with 180 rpm on seed culture medium containing glucose (20 g/L), yeast extract and 50% artificial seawater for 24 h. Artificial seawater composition was similar to that reported previously [36].

### 2.2. Batch Cultivation on Different Volatile Fatty Acids

The commercial VFAs formic acid (C1), acetic acid (C2), propionic acid (C3), butyric acid (C4), caproic acid (C5) and valeric acid (C6) were purchased from Sigma Aldrich (St. Louis, MO, USA). All VFAs were used as sole carbon source without any further sugar addition. Initially, 2 g/L of each VFA were mixed with an appropriate amount of yeast extract to maintain a C/N ratio of 10, followed by addition of 50% v/v artificial seawater. Tris (hydroxymethyl) aminomethane was used as buffer at a concentration of 100 mM. The volume was adjusted to 90% of the final volume and the pH was set to 6.8 with 3 M NaOH and 3 M HCl. Cultivation experiments were carried out in 250-mL Erlenmeyer flasks with 100 mL of working solution. Seed culture (10% v/v) was used to inoculate the medium and flasks were incubated in an orbital shaker with 180 rpm at 25 °C until stationary phase was reached. Growth was monitored by taking optical density readings at 680 nm (OD680) with a UV-Vis spectrophotometer (Spectra Max M2; Molecular Devices, San Jose, CA, USA). For VFAs that *Aurantiochytrium* sp. T66 could grow on, the concentration was gradually increased up to the point where the strain was not able to consume it. The C/N ratio for each trial was adjusted to 10 with yeast extract.

### 2.3. Morphologic Analysis and Estimation of Lipid Accumulation by Fluorescence Microscopy

Aliquots of 1 mL were harvested from the culture at early stationary phase, centrifuged and resuspended in 100 µL of 0.9% saline solution. 4,4-difluoro-1,3,5,7,8-pentamethyl-4-bora-3a,4a-diaza-s-indacene (BODIPY_493/503_) stock solution was prepared in DMSO (0.1 mg/mL) and 2 μL of it was added to the 100-µL samples. The samples were incubated for 5 min in the dark and imaging was carried out on a digital inverted fluorescence microscope equipped with a GFP light cube (EVOS-FL, (Thermo Fisher Scientific, Waltham, MA, USA). Cell size was determined by ImageJ 1.8.0 software (NIH, Bethesda, MD, USA).

### 2.4. Cell Dry Weight (CDW) Determination

At the end of each cultivation the cells were harvested to determine the cell dry weight. The culture was harvested into 50-mL tubes as soon as it reached stationary phase, as determined by daily OD680 measurements and centrifuged at 8000 rpm (7881× *g*) for 10 min in an Eppendorf 5804 R (Eppendorf Nordic A/S, Sverige Filial, Gulbackegatan, Malmö, Sweden) centrifuge with an F-34-6-38 rotor. Next, 2 mL of the supernatant was used to determine the residual carbon source (see Section 2.5). The cell pellet was washed three times with deionized (DI)-water, resuspended in DI-water, and finally pipetted into a preweighed metallic pan. The pans were dried in an oven at 50 °C until constant weight was attained. Cell dry weight was then determined gravimetrically.

### 2.5. Total Lipid Determination

To determine the amount of lipids in the cells, the dried biomass was milled with a mortar and pestle into a fine powder according to our previous report [37]. The powder was blended with chloroform:methanol (2:1, v/v) and incubated for 2 h under shaking. Subsequently, DI-water was added to the slurry at a volume equal to the volume of the slurry. The mixture was mixed thoroughly and afterwards centrifuged at 8000 rpm (7881× *g*) for 10 min. The bottom clear phase was aspirated into a preweighed glass plate and placed into a hot-air oven at 50 °C to evaporate the solvent. Lipid weight was then determined gravimetrically, and lipids were stored in a freezer at −20 °C until further analysis.

### 2.6. Residual Carbon Estimation by High-Performance Liquid Chromatography (HPLC)

Residual carbon source concentration was determined at the end of the cultivation by high-performance liquid chromatography (HPLC) analysis of the supernatant. Samples were prepared by filtration through a 0.2-μm syringe filter (Sartorius^TM^ Minisart^TM^ RC) into HPLC vials. A PerkinElmer (Waltham, Massachusetts, USA) Series 200 HPLC instrument equipped with a Bio-Rad Aminex HPX-87H Column (#1250140) was used to analyze C1, C2, C3, C4 and glucose. The analysis was programmed for 30 min and the column temperature was set to 65 °C with 5-mM H_2_SO_4_ as mobile solvent. A refractive index (RI) detector was used. Determination of residual C5 and C6 concentrations was performed as described above, with the difference that analysis time was extended to 50 min and the mobile phase was 90% v/v 5-mM H_2_SO_4_ and 10% v/v acetonitrile. Detection took place with a UV-Vis detector PerkinElmer (Waltham, Massachusetts, USA) set to 210 nm.

### 2.7. Analysis of Fatty Acid Profile by Gas Chromatography-Mass Spectrometry (GC-MS)

Lipids were transesterified as described by Wychen et al. (2013) [38] with some modifications. Specifically, a 50 to 100 mg lipid sample was dissolved in 2 mL of chloroform:methanol solution (2:1, v/v) in a screw-capped glass vial and 3 mL of 0.6 M HCl:methanol was added to it. The liquid was mixed and placed in a preheated water bath at 85 °C for 1 h. After that, it was cooled at room temperature to avoid extensive evaporation. The cooled liquid was transferred into 50-mL tubes and 3 mL HPLC-grade hexane was added. The tubes were mixed well and centrifuged at 8000 rpm (7881× *g*) for 10 min. The upper hexane layer was transferred to new glass vials. The fatty acid profile was determined by GC (Clarus 690; PerkinElmer) with MS (Clarus SQ8; PerkinElmer) using a capillary column (Elite 5MS; 30 m, 0.25 mm ID, 0.25 µm df, Cat. # N9316282; PerkinElmer). The oven was programmed to 50 °C for 0.5 min; temperature was then ramped to 194 °C at 30 °C/min for 3.5 min and then to 240 °C at 5 °C/min, where it was held for 10 min. The injection port temperature was adjusted to 250 °C and 1 µL of sample was injected with He as carrier gas in split mode (10:1). Solvent delay time was 3 min. The MS transfer line temperature was adjusted to 250 °C with 170 °C source temperature. Mass spectra (mass range 50 to 400 m/z) were recorded at 3 scans/s with electron ionization at 70 eV. Individual fatty acid methyl ester (FAME) peaks were identified by their mass spectra and further confirmed by a standard (Supelco 37 Component FAME Mix: 47885-U; Sigma-Aldrich (St. Louis, MO, USA). DHA concentration (g/L) and yield (mg/g_CDW_) were estimated by the following equations:

$$\mathrm{DHA}\;\mathrm{concentration}\;\left(\frac{g}{L}\right)=\frac{Total\;lipid\;concentration\;\left(g/L\right)\times \%\;of\;DHA\;in\;total\;lipid}{100}$$

DHA yield (mg/gCDW)=DHA concentration (mg/L)CDW (g/L)

### 2.8. Statistical Analysis

All experiments in this study were conducted in triplicate. All data are expressed as a mean ± standard deviation. The experimental data were subjected to one-way analysis of variance (ANOVA) as implemented in the Graph Pad Prism 8.4.2, 676 (GraphPad Software, San Diego, CA, USA) statistics platform. Tukey simultaneous tests were conducted to determine the statistical differences between the results obtained with various carbon sources. To ascertain that the observed variations were statistically significant, the probability (*p*) values were determined. A 95% confidence level (*p* < 0.05) was applied for all analyses.

## 3. Results and Discussion

### 3.1. Batch Cultivation of Aurantiochytrium sp. T66 on Different Volatile Fatty Acids

**Figure 1:**
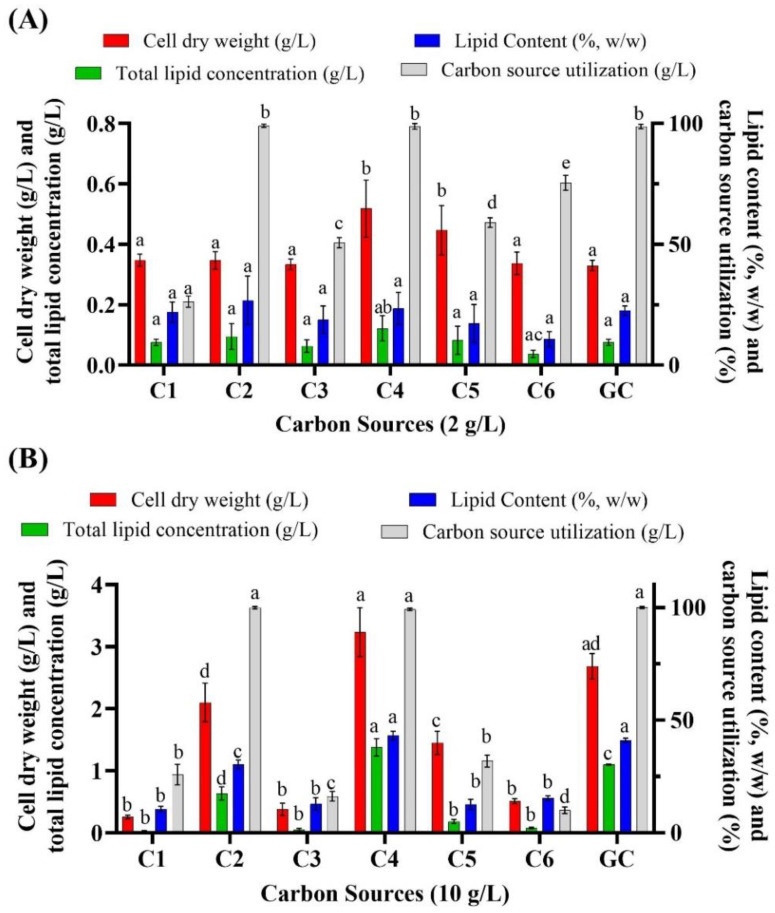
Batch cultivation of *Aurantiochytrium* sp. T66 on various volatile fatty acids (VFAs) at 2 g/L (**A**) and 10 g/L (**B**). Glucose (GC) was used as control. Error bars express the standard deviation of the mean (*n* = 3). Different letters (a,b,c,d,e) above the bars in each group of results indicate significant difference (*p* < 0.05), based on one-way ANOVA with Tukey’s test.

In the present study, the marine oleaginous thraustochytrid *Aurantiochytrium* sp. T66 was grown on artificial medium containing various concentrations of VFAs. When *Aurantiochytrium* sp. T66 was cultivated in 2 g/L of C1, C2, C3, C4, C5 and glucose (Figure 1A), cell dry weight was 0.35 g/L, 0.35 g/L, 0.34 g/L, 0.52 g/L, 0.45 g/L, 0.34 g/L and 0.33 g/L, respectively. The corresponding total lipid concentration was 0.07 g/L, 0.10 g/L, 0.06 g/L, 0.12 g/L, 0.08 g/L, 0.04 g/L and 0.08 g/L (Figure 1). Total carbon source consumption was complete only when cultivations were carried out in C2, C4 and glucose. The value of 2 g/L was chosen because it had been reported as the upper limit to avoid inhibition in other oleaginous microorganisms. One of them, the heterotrophic microalga *Chlorella protothecoides* (UTEX 25), exhibited optimal growth at 2 g/L VFAs (acetic acid:propionic acid:butyric acid; 6:1:3), resulting in 0.58 g/L cell dry weight and 0.278 g/L lipids [32]. Ramping the concentration of VFAs from 2 g/L to 4 g/L, severely inhibited growth and lipid accumulation by this microalga, which dropped to 0.33 g/L and 0.136 g/L, respectively [32]. In an effort to identify whether *Aurantiochytrium* sp. T66 could tolerate higher VFA concentrations, 10 g/L of carbon source was used (Figure 1B). Cell dry weight was now 0.25 g/L, 2.10 g/L, 0.38 g/L, 3.23 g/L, 1.46 g/L, 0.51 g/L and 2.68 g/L in C1, C2, C3, C4, C5 and glucose, respectively. The respective lipid content was 10.57%, 30.32%, 12.90%, 43.11%, 12.60%, 15.45% and 41.13% (Figure 1B).

The above findings showed that C2 and C4 were suitable substrates, with C4 in particular yielding better results than glucose. Almost complete consumption of C2 and C4 was observed at the end of cultivation. In contrast, thraustochytrids cultivated on C1, C3 and C6 exhibited growth inhibition and final cell dry weights below 1 g/L at both 2 g/L and 10 g/L of the substrate (Figure 1). Growth on C5 was partially inhibited and C5 consumption was 31.85%.

**Figure 2:**
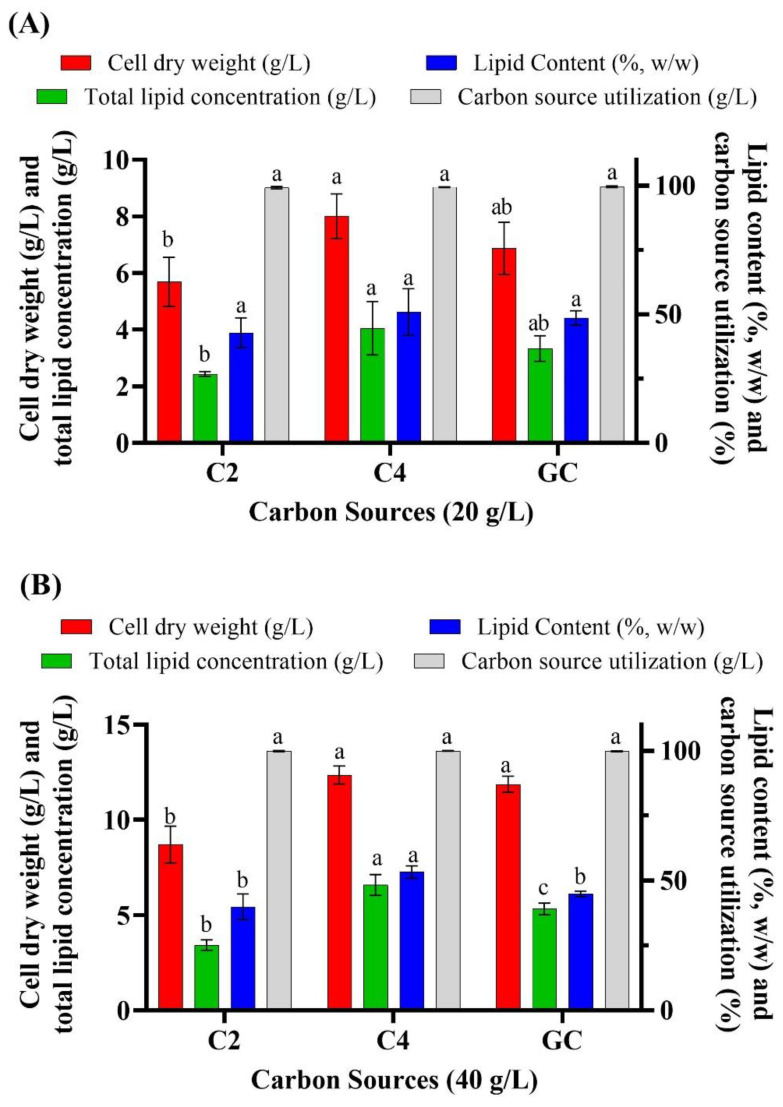
Batch cultivation of *Aurantiochytrium* sp. T66 on acetic acid (C2) and butyric acid (C4) at 20 g/L (**A**) and 40 g/L (**B**). Glucose (GC) was used as control. Error bars express the standard deviation of the mean Error bars express the standard deviation of the mean (*n* = 3). Different letters (a,b,c) above the bars in each group of results indicate significant difference (*p* < 0.05), based on one-way ANOVA with Tukey’s test.

Based on the results obtained so far, the most suitable VFAs appeared to be acetic acid (C2) and butyric acid (C4); hence, their concentration was increased to 20 g/L (Figure 2A). This resulted in a cell dry weight of 5.68 g/L, 8.00 g/L and 6.87 g/L when the microorganism was cultivated on C2, C4 and glucose, respectively, and a corresponding lipid concentration of 2.44 g/L, 4.05 g/L and 3.34 g/L. Because the cells showed no growth inhibition under these conditions, the concentration of VFAs was further increased to 40 g/L (Figure 2B). The highest cell dry weight (12.35 g/L) and total lipid concentration (6.59 g/L) was obtained with C4, followed by glucose (11.87 g/L and 5.34 g/L, respectively), whereas C2 resulted in lower biomass and lipid accumulation. Further increasing the concentration of VFAs (C2 and C4) to 60 g/L resulted in growth inhibition, which was not observed with 60 g/L of glucose (data not shown). Notably, 40 g/L of VFAs is higher than what was reported as tolerable by other oleaginous microorganisms. However, no study was reported to utilize VFAs as a sole carbon source by thraustochytrids. Among various types of oleaginous microorganisms only the oleaginous microalgae *Crypthecodinium cohnii* could utilize a high concentration of acetic acid and butyric acid as sole carbon sources for PUFA production. When Chalima et al. (2019) cultivated *C. cohnii* on various VFAs at an initial concentration of 5 to 50 g/L, the highest amount of biomass was obtained on 30 g/L of acetate (~6 g/L), 10 g/L of propionate (~3.8 g/L) and 15 g/L of butyrate (~3.9 g/L), while further increasing the concentration of substrates inhibited microbial growth [35]. Likewise, Ratledge et al. (2001) suggested that *C. cohnii* could grow on up to 1–16 g/L sodium acetate as a sole carbon source in a pH auxostat with acetate used to control the pH of the medium [39]. In another study, the freshwater microalga *C. protothecoides* was cultivated heterotrophically in waste activated sludge whose total chemical oxygen demand was 3840 mg/L, including acetic acid (1.2 g/L), propionic acid (0.45 g/L), butyric acid (0.23 g/L), isobutyric acid (0.24 g/L), valeric acid (0.36 g/L) and isovaleric acid (0.14 g/L) [40]. Waste activated sludge yielded a cell dry weight of 0.34 g/L; which was only slightly lower than that obtained on 15 g/L of pure glucose (0.63 g/L) or waste activated sludge enriched with selenite (0.50 g/L) [40]. Another freshwater microalga, *Chlorella sorokiniana*, was also heterotrophically cultivated on acetate, butyrate and a mixture of butyrate and acetate, but the concentration of these substrates was considerably lower (500-mM acetate and butyrate) than in the present study [41].

**Figure 3:**
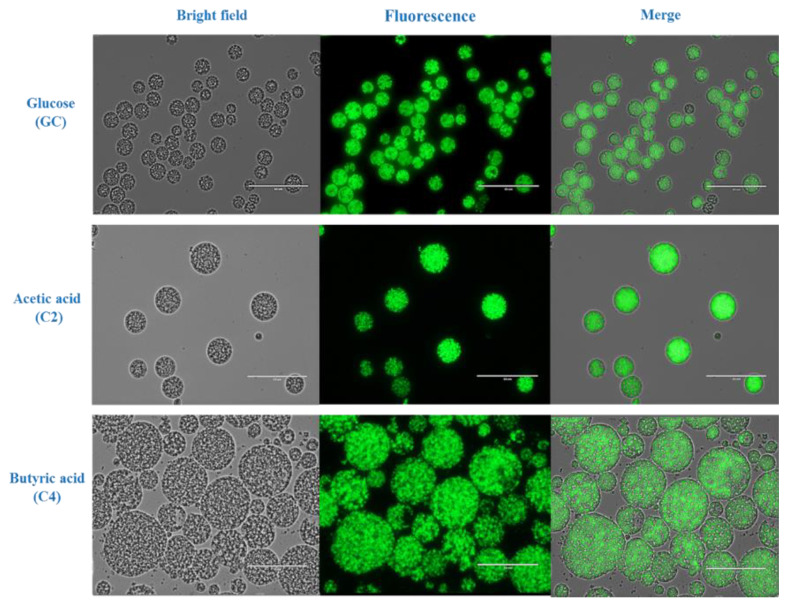
Morphologic analysis of cells and lipid droplets of *Aurantiochytrium* sp. T66 grown on 40 g/L of acetic acid, butyric acid and glucose. The cells were stained with 4,4-difluoro-1,3,5,7,8-pentamethyl-4-bora-3a,4a-diaza-s-indacene (BODIPY_493/503_) and observed by live fluorescence microscopy. Scale bars corresponds to 50 µm.

Cells grown on glucose, acetic acid and butyric acid were observed under a fluorescence microscope. The cells cultivated on butyric acid had a significantly larger size (41.23 ± 6.75 µm) and more lipid droplets compared to those cultivated on acetic acid, which was of intermediate size (20.09 ± 3.38 µm), and those grown on glucose, which were significantly smaller (11.11 ± 2.31 µm) (Figure 3). The cell number were high in the case of cells grown in glucose than the C2 and C4 cultivated cells while the lipid droplets per cell were high in the case of C4 grown cells. The largest cells were estimated to be almost 40 to 45 µm in diameter and, to the best of our knowledge, this is the largest oleaginous microorganism ever reported. While the exact mechanism for such a dramatic increase in size remains unknown, a possible reason may be the profound effect of butyric acid on metabolic flux and fatty acid synthesis [42].

### 3.2. Effect of Various Volatile Fatty Acids on the Fatty Acid Profile of Aurantiochytrium sp. T66

According to a literature survey by Morabito et al. (2019), more than 65% w/w of total lipids accumulated by thraustochytrids is made of C16:0 (palmitic acid) and C22:6 (DHA), which corresponds to one of the highest DHA contents among PUFA-producing microorganisms [43]. The total amount of lipids and their profile varies among thraustochytrid species, with the genera *Aurantiochytrium*, *Thraustochytrium* and *Schizochytrium* considered the most productive for DHA [44].

In the present study, when *Aurantiochytrium* sp. T66 was cultivated on 2 g/L of VFAs, it synthesized mainly C 14:0, C14:1, C15:0, C16:0, C16:1, C17:0, C18:0 and C18:1 (Table 1). Long-chain PUFAs (>18:3), including DPA and DHA, were absent from the total lipids, which may be due to insufficient amounts of desaturases and elongases at very low substrate concentrations. Two different pathways exist for PUFA synthesis: the oxygen-dependent aerobic fatty acid synthase pathway (FAS), also known as elongase–desaturase pathway, and the oxygen-independent anerobic polyketide synthase pathway (PKS)—also designated as the PUFA synthase pathway [45]. In the FAS pathway, the starting point for PUFA synthesis is C18:3–9,12,15, which is converted by Δ6 desaturase into C18:4–6,9,12,15 and by elongase into C20:4–8,11,14,17. The latter is then converted into C20:5–5,8,11,14,17 by Δ5 desaturase, followed by elongation to 22:5–7,10,13,16,19 and, finally, conversion into DHA (22:6–4,7,10,13,16,19) by Δ4 desaturase [43,45,46,47]. Hence, PUFA synthesis through the FAS pathway can only proceed with an adequate supply of C18:3 as a substrate. Insufficient C18:3 may explain the poor production of DHA at low concentrations of VFAs. However, in the PKS pathway, acyl carrier protein (ACP), generated by CoA, is used as a covalent attachment point for chain synthesis, which proceeds with reiterative cycles. During the full fatty acid synthesis process, a series of enzymes including 3-ketoacyl synthase (KS), 3-ketoacyl-ACP reductase (KR), enoyl reductase (ER), and dehydrase/isomerase (DH) are necessary [48]. However, the whole genome annotation results showed that *S. limacinum* does not contain Δ4 desaturase, in agreement with literature reports. On the other hand, some ORFs in *S. limacinum* were predicted to be potential PKS proteins [48]. This means *S. limacinum* could not synthesize DHA by the FAS route, but rather employs the PKS system for PUFA biosynthesis [45,49,50]. Contrasting to this, some thraustochytrids such as *Thraustochytrium* sp., synthesize DHA via Δ4 desaturation where desaturase introduces Δ4 bond into 22:5(n−3) that resulted into DHA [51,52,53].

When cultivation was shifted from 2 to 10 g/L of VFAs, the main fatty acids were C14:0, C15:0, C16:0, C17:0, C18:0, C18:1, as well as long-chain fatty acids such as C20:4n6, DPA and DHA (Table 2). The DHA content (w/w_total lipids_) was 9.57%, 28.75%, 7.28%, 18.64%, 7.88%, 6.01% and 4.14% when cultivation was carried out in C1, C2, C3, C4, C5, C6 and glucose, respectively (Table 2). The highest DHA content was observed with C2 as substrate followed by C4. A possible reason is the direct production of acetyl-CoA, a key intermediate in fatty acid synthesis, from VFA via a non-conventional route [54]. When oleaginous microorganisms are grown on glucose as sole carbon source, the cells start assimilating glucose for glycolysis and form pyruvate, which is transported to mitochondria and fed into the tricarboxylic acid cycle. This yields citrate, which is transported back to the cytosol, where it is converted into acetyl-CoA by ATP:citrate lyase [55]. In contrast, when oleaginous cells assimilate acetic acid, this is converted directly into acetyl-CoA in the cytosol by acetyl-CoA synthetase in a single step [47,54].

**Figure 4:**
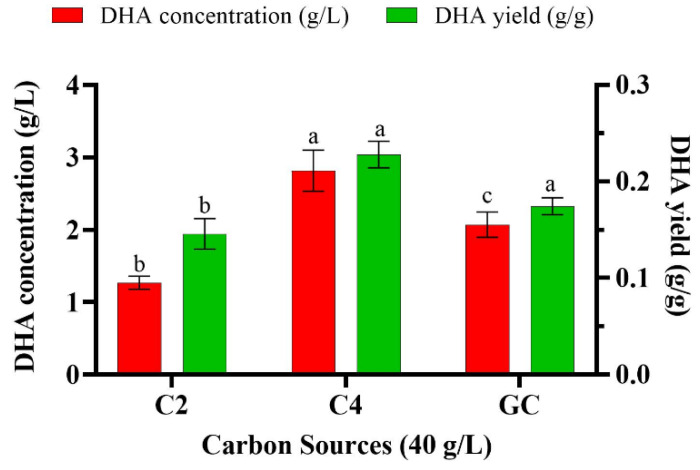
DHA concentration and yield of *Aurantiochytrium* sp. T66 cultivated in 40 g/L of C2, C4 or glucose. Error bars express the standard deviation of the mean (n = 3). Different letters (a,b,c) above the bars in each group of results indicate significant difference (*p* < 0.05), based on one-way ANOVA with Tukey’s test.

Finally, *Aurantiochytrium* sp. T66 was shifted to an even higher concentration of the substrate to study the effect on DHA production of 20 g/L and 40 g/L of C2 or C4. At 20 g/L of C2, C4 or glucose, DHA content increased to 30.40%, 36.25% and 29.58%, respectively (Table 3); the other major fatty acids being produced were C15:0, C16:0, C17:0, C18:0, C18:3n3 and DPA. When cells are cultivated on C2 compounds such as acetic acid, the tricarboxylic acid cycle cannot provide sufficient carbon skeleton building blocks, ATP and reducing power (NADH). This shortage prompts the induction of another metabolic pathway, the glyoxylate cycle, and the activation of enzymes, such as isocitrate lyase and malate synthase [56,57]. It has been suggested that the oleaginous microalgae *Chlorella vulgaris* can express the glyoxylate cycle enzymes even when cultivated on glucose, however they are not active unless the microorganism is grown on acetate [58]. The activity and concentration of isocitrate lyase was found to be increased in another microalga, *Scenedesmus obliquus*, after being cultivated on acetate in the dark; moreover, the concentration of this enzyme increased with increasing concentration of acetate [59]. In the present study, when cells were grown in 40 g/L C2 and C4, DHA content increased to 36.93% and 42.63%, respectively, while on glucose the DHA concentration was 38.72% (Table 4). The highest DHA concentration (2.81 g/L) and DHA yield (0.23 g/g_CDW_) were observed when cells were cultivated in 40 g/L of C4, followed by 40 g/L of glucose (2.07 g/L and 0.17 g/g_CDW_, respectively) (Figure 4). Addition of 1% v/v n-dodecane (C12) to the medium of the marine microalga *C. cohnii* was shown to enhance cell dry weight and lipid accumulation, including DHA content (51% w/w of total lipids) [60]. The authors suggested that the hydrocarbon in the medium acted as an oxygen vector and could improve oxygen availability to the microorganisms, thus enhancing DHA productivity. At the same time, the total content of unsaturated fatty acids (16:1n7, 18:1n9 and 22:5n3) remained unaffected, suggesting that the increase in DHA was independent of the oxygen-dependent desaturase [60].

## 4. Conclusions

The present study indicates that VFAs can be utilized efficiently as a sole carbon source for the cultivation of oleaginous marine thraustochytrids. Cell dry weight and lipid production were particularly high when *Aurantiochytrium* sp. T66 was cultivated on butyric acid as sole carbon source. The fatty acid profile of this strain revealed up to 42.63% of DHA in total lipids after cultivation on VFAs. The maximum DHA concentration (2.81 g/L) and DHA yield (0.23 g/g_CDW_) were attained when cells were cultivated in 40 g/L butyric acid, and were even higher than those observed in 40 g/L glucose (2.07 g/L and 0.17 g/g_CDW_, respectively). The important finding of this study was the dramatic increment in cell size after cultivation on 40 g/L of butyric acid that provides a foundation for the further exploration of the assimilation pathway and metabolic flux in thraustochytrids. Moreover, replacing costly refined sugars as carbon source with VFAs derived from abundant waste streams will make microbial PUFA synthesis more economical at an industrial scale.

## Figures and Tables

**Table 1:** Fatty acid composition (wt% of total fatty acids) of *Aurantiochytrium* sp. T66 cultivated on 2 g/L of various VFAs (C1:0, formic acid; C2:0, acetic acid; C3:0, propionic acid; C4:0, butyric acid; C5:0, valeric acid and C6:0, caproic acid). Glucose (GC) was used as control.

Fatty Acids (%) in Total Lipid	C1	C2	C3	C4	C5	C6	GC
**C14:0**	6.04 ± 0.32 ^b^	7.50 ± 0.28 ^c^	7.26 ± 0.12 ^c,d^	6.46 ± 0.57 ^b,c^	5.44 ± 0.16 ^b^	6.90 ± 0.52 ^b,c^	21.59 ± 0.30 ^a^
**C14:1**	12.12 ± 0.58 ^b^	14.89 ± 0.63 ^c^	17.60 ± 0.81 ^d^	17.30 ± 0.41 ^d^	12.64 ± 0.42 ^b^	25.93 ± 1.14 ^a^	ND
**C15:0**	ND	ND	ND	ND	ND	ND	18.60 ± 0.73
**C15:1**	3.67 ± 0.61 ^b^	3.42 ± 0.42 ^b^	4.14 ± 0.13 ^b^	4.44 ± 0.65 ^b^	3.09 ± 0.23 ^b^	8.38 ± 0.34 ^a^	ND
**C 16:0**	35.44 ± 0.82 ^b^	37.72 ± 0.94 ^c^	32.90 ± 1.23 ^f^	38.46 ± 0.60 ^c,d^	36.44 ± 0.73 ^b,c^	32.63 ± 0.42 ^e,f^	44.31 ± 0.63 ^a^
**C16:1**	8.54 ± 1.17 ^a^	8.17 ± 0.64 ^a^	8.18 ± 0.82 ^a^	7.97± 0.35 ^a^	8.68 ± 1.00 ^a^	7.73 ± 0.92 ^a^	ND
**C 18:0**	18.66 ± 0.92 ^b^	22.45 ± 0.58 ^c^	25.58 ± 1.08 ^d^	23.23 ±1.19 ^c,e^	27.79 ± 0.24 ^a,d^	17.736 ± 0.89 ^b^	9.64 ± 0.35 ^f^
**C18:1**	14.88 ± 1.40 ^d^	5.75 ± 0.48 ^a,b^	4.37 ± 0.73 ^a,b^	2.16 ± 0.41 ^b,c^	5.81 ± 0.89 ^a^	ND	ND
**DPA**	ND	ND	ND	ND	ND	ND	2.65 ± 0.37
**DHA**	ND	ND	ND	ND	ND	ND	2.48 ± 0.52

ND = not detected; Results are presented as mean ± SD, *n* = 3. Different superscript small letters (a,b,c,d,e,f) of each fatty acid obtained with various carbon sources indicate significant difference (Tukey’s test, *p* < 0.05).

**Table 2:** Fatty acid composition (wt% of total fatty acids) of *Aurantiochytrium* sp. T66 cultivated on 10 g/L of various VFAs (C1:0, formic acid; C2:0, acetic acid; C3:0, propionic acid; C4:0, butyric acid; C5:0, valeric acid and C6:0, caproic acid). Glucose (GC) was used as control.

Fatty Acids (%) in Total Lipid	C1	C2	C3	C4	C5	C6	GC
C14:0	6.92 ± 0.42 ^b^	4.79 ± 0.32 ^c^	9.00 ± 0.40 ^d^	15.08 ± 0.37 ^a^	7.74 ± 0.35 ^b,d^	5.09 ± 0.30 ^c^	14.99 ± 1.13 ^a^
C14:1	ND	ND	ND	ND	ND	17.37 ± 0.72	ND
C15:0	18.54 ± 0.49 ^d^	3.49 ± 0.03 ^c^	19.59 ± 0.90 ^a^	10.25 ± 0.28 ^b^	22.56 ± 1.28 ^a^	8.39 ± 0.82 ^b^	21.37 ± 0.85 ^a^
C 16:0	28.43 ± 0.85 ^b^	23.22 ± 0.32 ^c^	29.58 ± 0.82 ^b^	34.19 ± 1.25 ^d^	30.19 ± 1.05 ^b^	31.29 ± 1.01 ^b,e^	42.79 ± 0.47 ^a^
C16:1	ND	ND	ND	ND	ND	5.10 ± 0.49	ND
C17:0	9.22 ± 0.69 ^a^	ND	9.27 ± 0.45 ^a^	5.77 ± 0.55 ^b^	8.92 ± 0.38 ^a^	ND	8.56 ± 0.93 ^a^
C 18:0	14.44 ± 0.75 ^a^	8.90 ± 0.52 ^b^	14.89 ± 1.37 ^a^	6.74 ± 0.49 ^c^	14.32 ± 0.55 ^a^	8.63 ± 0.78 ^b^	5.79 ± 0.33 ^c^
C18:1	ND	1.84 ± 0.49	ND	ND	ND	9.46 ± 0.89	ND
C20:0	ND	ND	ND	ND	ND	5.58 ± 0.81	ND
C20:4	2.31 ± 0.27 ^b^	5.13 ± 0.57 ^a^	1.03 ± 0.14 ^c^	ND	0.96 ± 0.05 ^c,d^	ND	ND
EPA	2.32 ± 0.16 ^b^	5.24 ± 0.05 ^a^	1.60 ± 0.27 ^c^	ND	1.94 ± 0.14 ^b,c^	ND	ND
DPA	7.52 ± 0.60 ^b^	18.29 ± 0.78 ^a^	7.27 ± 0.78 ^b^	8.88 ± 0.41 ^b^	4.87 ± 0.41 ^d^	2.41± 0.12 ^c^	2.20 ± 0.13 ^c^
DHA	9.57± 0.68 ^b^	28.73 ± 1.28 ^a^	7.28 ± 0.21 ^c^	18.64 ± 0.59 ^d^	7.88 ± 0.54 ^b,c^	6.01 ± 0.53 ^c^	4.14 ± 0.28 ^c^

ND = not detected; Results are presented as mean ± SD, *n* = 3. Different superscript small letters (a,b,c,d,e) of each fatty acid obtained with various carbon sources indicate significant difference (Tukey’s test, *p* < 0.05).

**Table 3:** Fatty acid composition (wt% of total fatty acids) of *Aurantiochytrium* sp. T66 cultivated on 20 g/L of acetic acid (C2) or butyric acid (C4). Glucose (GC) was used as control.

Fatty Acids (%) in Total Lipid	C2	C4	GC
C14:0	2.85 ± 0.35 ^b^	6.67 ± 0.31 ^a^	8.33 ± 0.58 ^a^
C15:0	10.32 ± 0.40 ^a^	2.60 ± 0.18 ^b^	8.03 ± 0.10 ^c^
C 16:0	25.59 ± 0.43 ^a^	19.79 ± 0.77 ^b^	18.54 ± 1.04 ^b^
C16:1	5.48 ± 0.68	2.37 ± 0.35	ND
C 18:0	3.67 ± 0.17 ^b^	3.00 ± 0.53 ^b^	7.99 ± 0.40 ^a^
C18:1	ND	1.33 ± 0.16	ND
C20:4	1.74 ± 0.17	5.23 ± 0.53	ND
EPA	0.72 ± 0.13 ^b^	1.89 ± 0.48 ^a^	1.59 ± 0.43 ^a^
DPA	12.16 ± 0.14 ^c^	13.93 ± 0.48 ^b^	17.00 ± 0.62 ^a^
DHA	36.93 ± 0.69 ^a^	42.63 ± 1.08 ^b^	38.72 ± 1.04 ^a^

ND = not detected; Results are presented as mean ± SD, *n* = 3. Different superscript small letters (a,b,c) of each fatty acid obtained with various carbon sources indicate significant difference (Tukey’s test, *p* < 0.05).

**Table 4:** Fatty acid composition (wt% of total fatty acids) of *Aurantiochytrium* sp. T66 cultivated on 40 g/L of acetic acid (C2) or butyric acid (C4). Glucose (GC) was used as control.

Fatty Acids (%) in Total Lipid	C2	C4	GC
C14:0	7.65 ± 0.23 ^a^	2.45 ± 0.17 ^b^	5.31 ± 0.04 ^b^
C15:0	8.93 ± 0.28 ^b^	3.43 ± 0.18 ^c^	18.3 ± 0.76 ^a^
C 16:0	22.78 ± 0.46 ^a^	20.34 ± 0.48 ^b^	15.58 ± 1.02 ^c^
C17:0	7.11 ± 0.26 ^b^	1.02 ± 0.13 ^c^	11.74 ± 0.37 ^a^
C 18:0	2.45 ± 0.91 ^b^	4.47 ± 0.73 ^a^	4.043 ± 0.40 ^a,b^
C20:4	1.17 ± 0.14	5.28 ± 0.37	ND
EPA	1.65 ± 0.37 ^b^	3.12 ± 0.16 ^a^	0.82 ± 0.03 ^c^
DPA	17.94 ± 0.29 ^b^	23.35 ± 0.81 ^a^	14.54 ± 0.72 ^c^
DHA	30.40 ± 0.64 ^b^	36.25 ± 1.17 ^a^	29.58 ± 0.95 ^b^

ND = not detected; Results are presented as mean ± SD, *n* = 3. Different superscript small letters (a,b,c) of each fatty acid obtained with various carbon sources indicate significant difference (Tukey’s test, *p* < 0.05).
